# The involvement of miR-100 in bladder urothelial carcinogenesis changing the expression levels of mRNA and proteins of genes related to cell proliferation, survival, apoptosis and chromosomal stability

**DOI:** 10.1186/s12935-014-0119-3

**Published:** 2014-11-26

**Authors:** Denis R Morais, Sabrina T Reis, Nayara Viana, Camila Berfort Piantino, Cristina Massoco, Caio Moura, Nelson Dip, Iran A Silva, Miguel Srougi, Katia RM Leite

**Affiliations:** Laboratory of Medical Research, Department of Urology – LIM55, University of Sao Paulo Medical School, Sao Paulo, Brazil; Department of Pathology, University of Sao Paulo Veterinary Medicine and Zootechnics School, Sao Paulo, Brazil

**Keywords:** Bladder cancer, MicroRNA, miR-100, Gene expression, Protein expression, PCR, Western blotting

## Abstract

**Introduction:**

MicroRNAs (miRNA) are small non-coding RNAs that play an important role in the control of gene expression by inhibiting protein translation or promoting messenger RNA degradation. Today, miRNAs have been shown to be involved in various physiological and pathological cellular processes, including cancer, where they can act as oncogenes or tumor suppressor genes. Recently, lowered expression of miR-100, resulting in upregulation of FGFR3, has been correlated with low-grade, non-invasive bladder urothelial cancer, as an alternative oncogenesis pathway to the typical FGFR3 gene mutation. Our aim is to analyze the role of miR-100 in bladder cancer cell lines in controlling the expression of some of its possible target genes, including FGFR3 and its relationship with proliferation, apoptosis and DNA ploidy.

**Methods:**

The bladder cancer cell lines RT4 and T24 were transfected with pre-miR 100, anti-miR 100 and their respective controls using a lipid-based formulation. After transfection mRNA and protein levels of its supposed target genes THAP2, BAZ2A, mTOR, SMARCA5 and FGFR3 were analyzed by quantitative real time polymerase chain reaction (qRT-PCR) and western blotting. Cell proliferation, apoptosis and DNA ploidy were analyzed by flow cytometry. For statistical analysis, a *t*-test was applied, p < 0.05 was considered significant.

**Results:**

After miR-100 transfection, there was a significant reduction in the mRNA of mTOR (p = 0.006), SMARCA5 (p = 0.007) and BAZ2A (p = 0.029) in RT4, mTOR (p = 0.023) and SMARCA5 (p = 0.015) in T24. There was a reduction in the expression of all proteins, variable from 22.5% to 57.1% in both cell lines. In T24 miR-100 promoted an increase in cell proliferation and anti-miR 100 promoted apoptosis characterizing miR-100 as an oncomiR in this cell line representative of a high-grade urothelial carcinoma.

**Conclusion:**

miR-100 transfection reduces expression of BAZ2A, mTOR and SMARCA5 mRNA and protein in BC cell lines. miR-100 would be classified as an oncomiR in T24 cells representative of high grade urothelial carcinoma promoting increase in cell proliferation and reduction in apoptosis. The knowledge of miRNA role in tumors will allow their use as tumor markers and targets for new therapies.

## Introduction

MicroRNAs (miRNAs) are a class of small non-coding RNAs, approximately 19 to 22 nucleotides in length that are important in the regulation of gene expression [1]. At present, approximately 1,600 human miRNAs have been identified by sequence analysis (miRBase) [2], and it is believed that miRNAs regulate approximately 30% of all human genes [3]. Aberrant expression patterns and functional abnormalities of miRNAs are implicated in several human diseases, including cancer [4]. They play important roles in the regulation of cellular processes, such as proliferation, apoptosis and differentiation. Recent evidences suggest that miRNAs are context and cell-dependent and behave differently depending on the tumor type or tumor stage [4]. We have recently demonstrated that miR-100 is under-expressed in 100% of low-grade, non-invasive bladder cancers, with FGFR3 as a putative target gene [5], representing a possible first step of tumorigenesis, before the typical FGFR3 mutation. There are other proposed target genes of miR100 that could be involved in bladder carcinogenesis, such as THAP2, BAZ2A, SMARCA5 and mTOR. Our aim is to study the role of miR-100 in bladder cancer cell lines in controlling the expression of mRNAs and proteins of its putative target genes and its relationship with proliferation, apoptosis and DNA ploidy.

## Methods

### Cell culture

The human bladder cancer cell line RT4 was obtained from the European Collection of Cell Cultures (ECACC) and was cultured in McCoy’s medium supplemented with 20% fetal bovine serum with a 1% antibiotic/antimycotic solution (Sigma Co., St. Louis, MO, USA) at 37°C in an atmosphere of 5% CO_2_. The human bladder cancer cell line T24 was kindly provided by Prof. Daisy Salvadori (Unesp, Botucatu, SP, Brazil) and was maintained as described above.

### miR-100 and anti-miR 100 cell transfection

The cells were seeded in 24-well plates at a concentration of 5.0 × 10^4^/well for miRNA and anti-miRNA transfection. The reverse-transfections into the bladder cell lines were carried out in Opti-MEM I using cationic liposomes (Lipofectamine 2000; Invitrogen, Carlsbad, CA, USA) according to the manufacturer’s protocol. Synthetic miR-100 (Catalog number AM17100 – Assay ID PM 10188), anti-miR 100 (Catalog number AM17000 – Assay ID AM 10188) and their respective scrambled controls, control miR (ctrl miR; catalog number AM17110) and control anti-miR (ctrl αmiR; catalog number AM17010), were purchased from Ambion (Austin, TX, USA). The negative controls and the miRNAs were used at a final concentration of 50 nM.

### RNA extraction and quantitative real-time PCR (qRT-PCR)

The RNA extraction was performed using the mirVana kit (Ambion, Austin, TX, USA) according to the manufacturer’s instructions. RNA concentrations were determined by 260/280 nm absorbance using a Nanodrop® ND-1000 spectrophotometer (Thermo Scientific). For cDNA synthesis, 200 ng of total RNA was reverse transcribed using random primers and Multiscribe reverse transcriptase (Applied Biosystems, Foster City, CA, USA) with incubation at 25°C for 10 min, followed by 37°C for 120 min and 85°C for 5 min. To determine the expression of the mRNAs for THAP2, BAZ2A, mTOR, SMARCA5 and FGFR3, qRT-PCR was performed using TaqMan assays from Applied Biosystems (Foster City, CA, USA). All samples were amplified in a 7500 fast real-time PCR system (Applied Biosystems, Foster City, CA, USA). The samples were processed at 50°C for 2 min, followed by 95°C for 10 min and 40 thermal cycles of 95°C for 15 seconds and 60°C for 1 min. The reactions were conducted in duplicate, and human ß2 microglobulin was used as an internal endogenous control. The relative expression level of the mRNAs was calculated by the 2^-ΔΔCt^ method. The data shown are representative of three independent experiments.

### Western blotting

For western blotting, T24 and RT4 cells (2,5 × 10^5^ cells/well) were transfected with 50 nM of pre-miR 100, anti-miR 100, or their respective controls. After 48 h, the cells were resuspended and lysed in their wells using RIPA buffer and protease inhibitor (Millipore, Billerica, MA, USA). Protein concentrations were established using the Pierce 660 nm Protein Assay (Thermo Scientific). From the cell lysate, 30 μg of protein was electrophoresed on a 12% SDS-polyacrylamide gel. After electrophoresis, the protein was transferred to a PVDF Immobilon-P membrane (Millipore, Billerica, MA, USA). The membrane was blocked in Tris-buffered saline (TBS) containing 1% BSA (bovine serum albumin) (Sigma, St. Louis, MO, USA) and 1% Tween-20 for 15 sec at room temperature. The 20-min primary antibody incubation occurred in a SNAP camera (Millipore) following the manufacturer’s instructions. The primary antibodies used were: mTOR (Abcam 1:500), FGFR3 (Abcam 1:600), SMARCA5 (Abcam 1:2000), BAZ2A (Abcam 1:500) and THAP2 (Proteintech 1:600). β-actin (Millipore 1:500) was used as a control. The secondary antibody was used at a 1:5000 dilution with an incubation time of 20 min (Millipore) in the same equipment. Protein expression analysis was performed using Alliance 4.7 equipment (Uvitec, Cambridge, UK) running the software package Alliance 16.06.

### Analysis of DNA ploidy, cell cycle and apoptosis by flow cytometry

For analysis of DNA ploidy, cell cycle and apoptosis the cells T24 and RT4 were transfected with pre-miR 100, anti-miR 100 and their respective controls as described above, and after 96 hours of transfection, cells were fixed in 70% ethanol, stained with propidium iodide (PI) diluted in PBS (200 μg/ml, RNAse A 20 mg/ml, triton X-100 and PBS pH 7.4) for 15 min at 37°C. The apoptosis was measured using the Annexin V Apoptosis Detection Kit I (BD Biosciences, USA) according the instructions of the manufacturer’s. RT4 and T24 cells (1 × 10^5^ cells per well) were seeded in a 12-well plate and after 96 hours of transfection they were harvested and stained with annexin-V-FITC and PI. The analyses of DNA ploidy, cell cycle and apoptosis were performed using the FlowJo (Tree Star) version 10 and FlowJo version 7.6.1 (Three Star). The results were expressed as mean ± SEM.

### Statistical analysis

For statistical analysis, experiments were performed in triplicate. With GraphPad Prism version 5 software Student’s *t-*test was used to calculate the difference in expression of target genes, cell proliferation and apoptosis, and p < 0.05 was considered significant.

## Results

**Figure 1:**
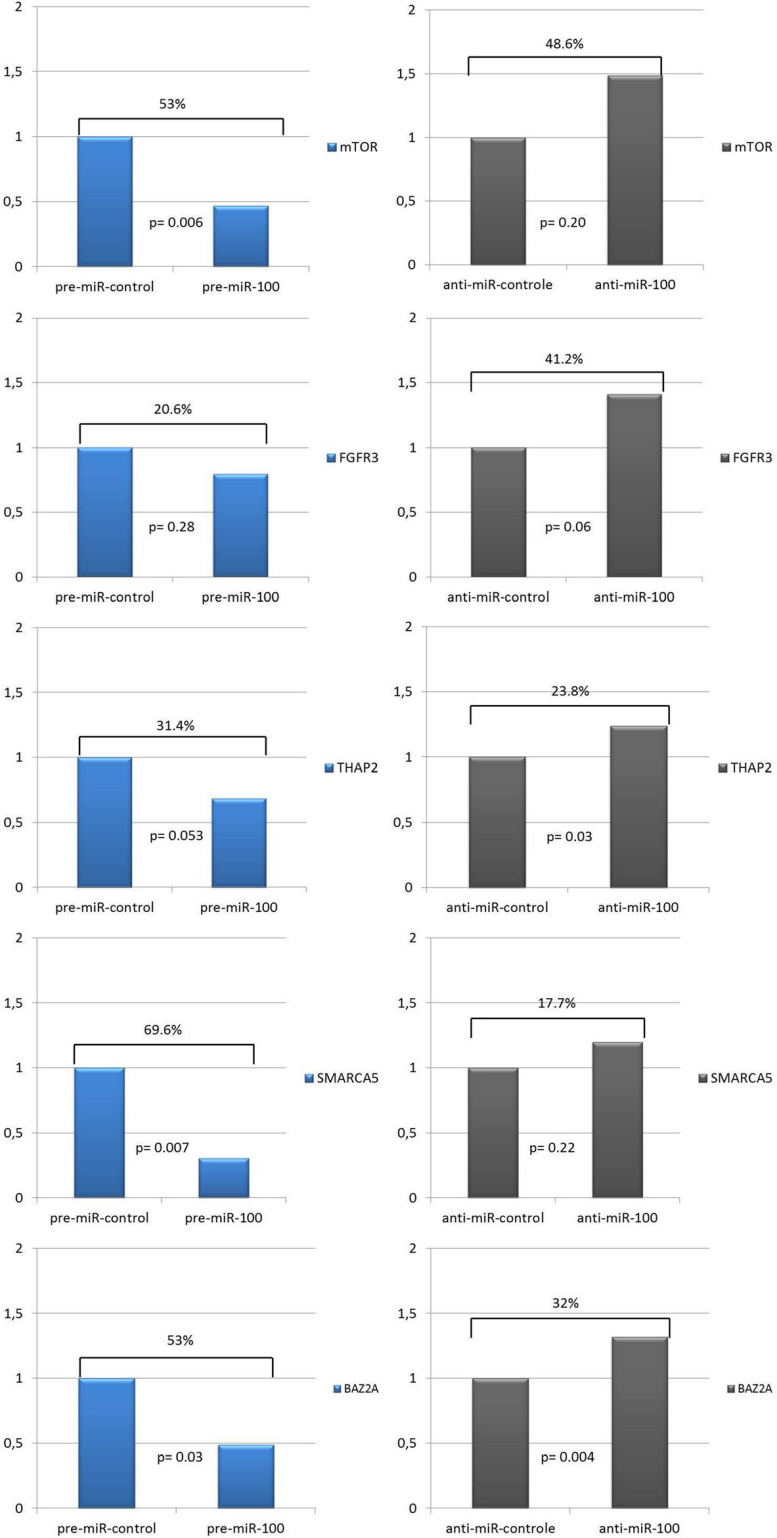
**mRNA expression of FGFR3, mTOR, BAZ2A, SMARCA5 and THAP2 after transfection of the RT4 low grade urothelial bladder cancer cell line with miR-100 and anti-miR 100.**

RT4 cells exposed to pre-miR 100 showed a significant reduction in mRNA expression of mTOR (53%, p = 0.006), SMARCA5 (69.6%, p = 0.007) and BAZ2A (53%, p = 0.029). THAP2 reduction could be considered marginal (31.4%, p = 0.053). FGFR3 mRNA expression decreased 20.6% but did not reach statistical significance compared to the control (p = 0.28). When exposed to anti-miR 100, we observed an increase in the expression of all genes, but the increase was significant only for THAP2 and BAZ2A, with increases of 23.8% (p = 0.03) and 32% (p = 0.004), respectively (Figure 1).

**Figure 2:**
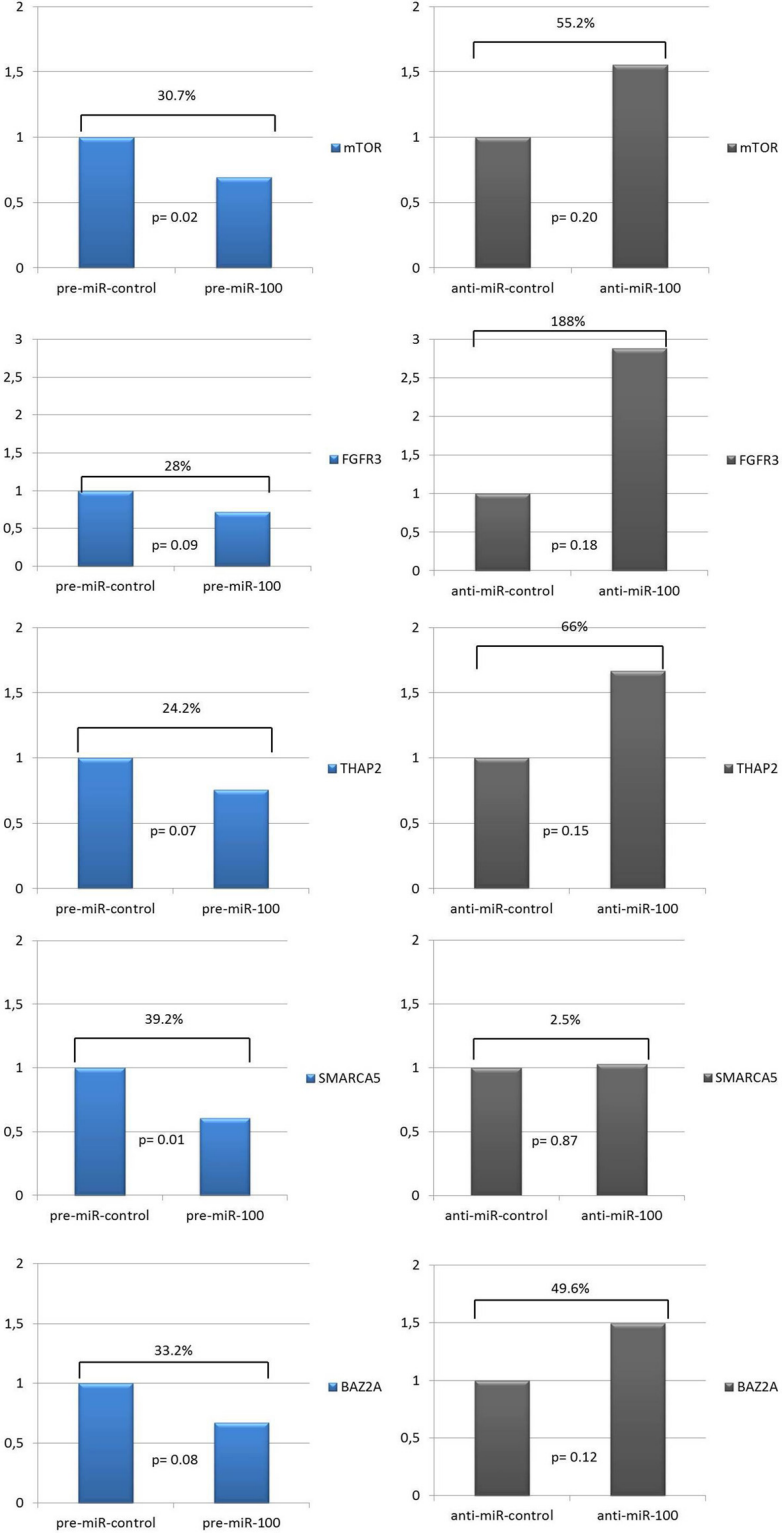
**mRNA expression of FGFR3, mTOR, BAZ2A, SMARCA5 and THAP2after transfection of the T24 high grade urothelial bladder cancer cell line with miR-100 and anti-miR 100.**

T24 cells showed a significant reduction in the expression of mTOR (30.7%, p = 0.02) and SMARCA5 (39.2%, p = 0.01) after exposure to pre-miR 100. The other four genes also showed decreased, but non-significant, expression relative to the controls. When exposed to anti-miR 100, all genes except SMARCA5 showed enhanced, but non-significant, expression relative to the controls (Figure 2).

**Figure 3:**
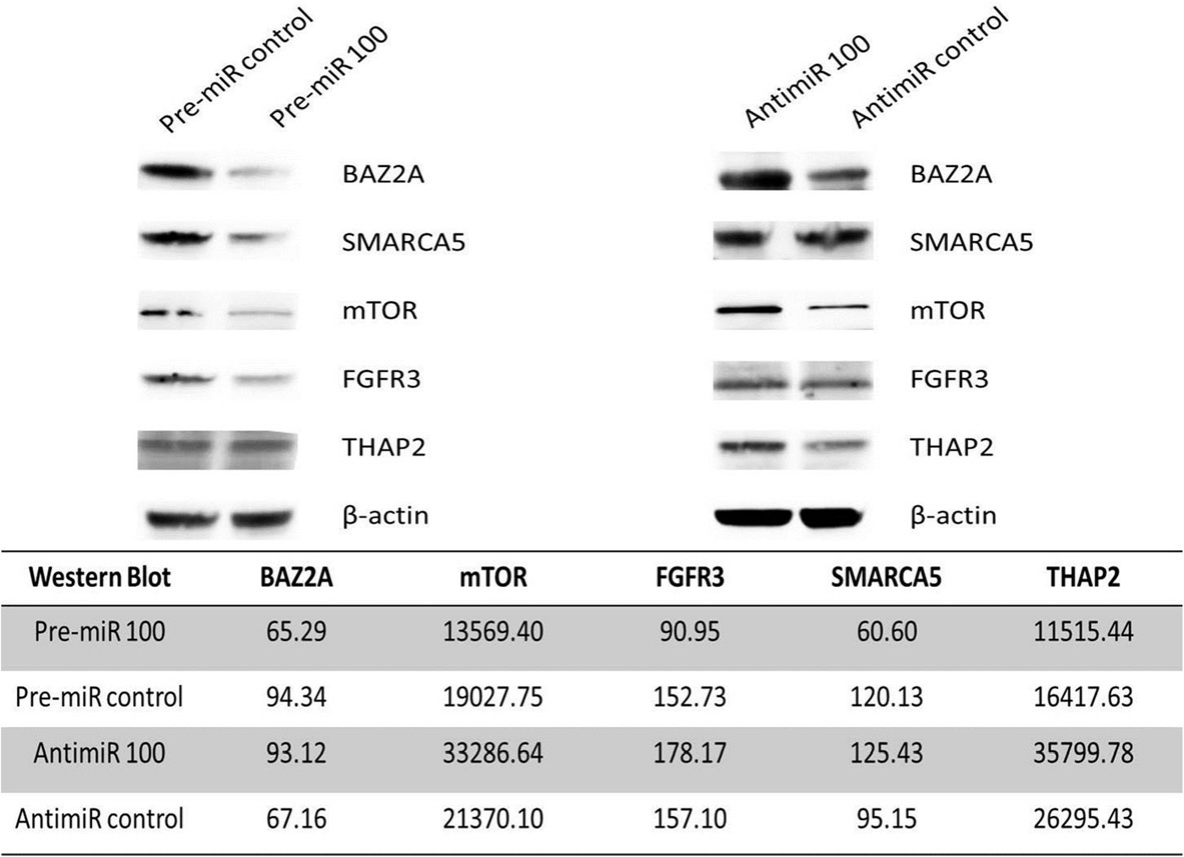
**mTOR, FGFR3, THAP2, SMARCA5 and BAZ2A protein expression in RT4 bladder cancer cells, evaluated by western blotting, after exposure to miR-100.**

**Figure 4:**
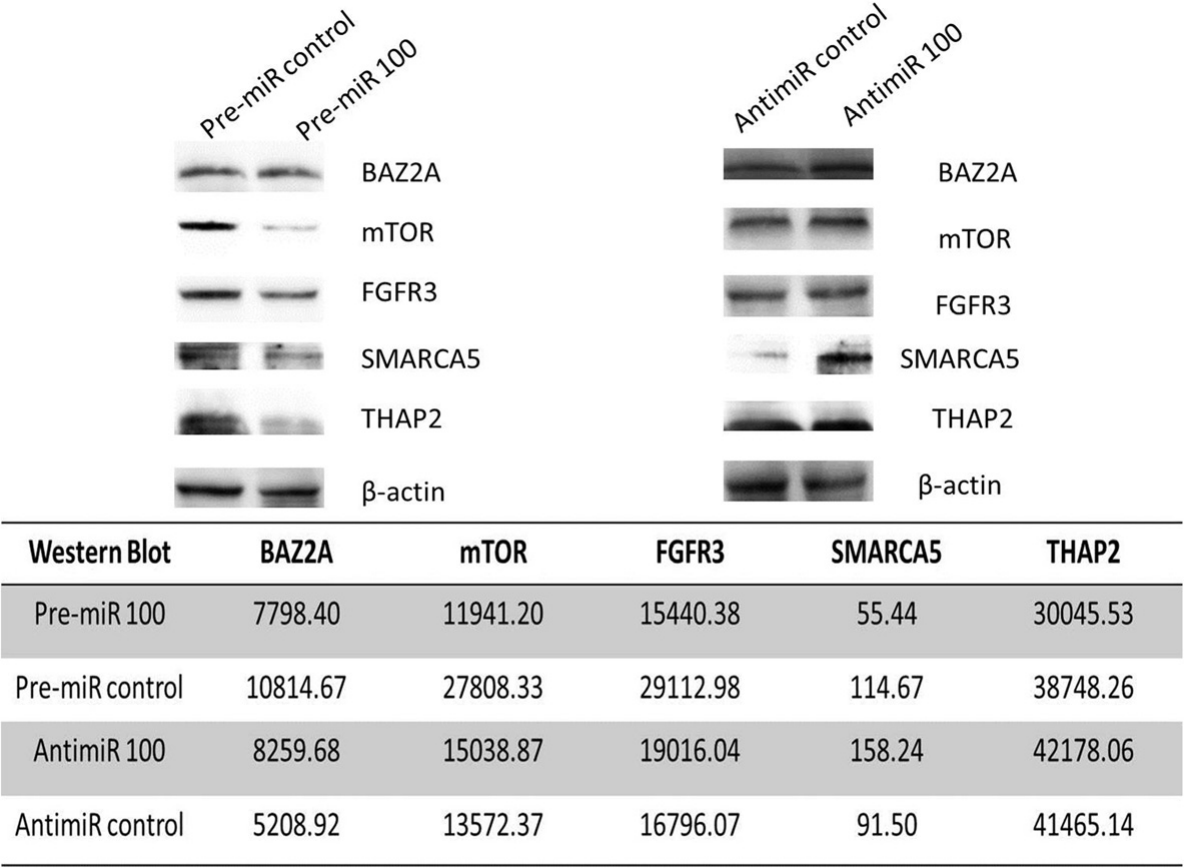
**mTOR, FGFR3, THAP2, SMARCA5 and BAZ2A protein expression in T24 bladder cancer cells, evaluated by western blotting, after exposure to miR-100.**

The western blot analysis of the proteins encoded by the genes THAP2, BAZ2A, mTOR, SMARC5 and FGFR3 in RT4 and T24 bladder cancer cells exposed to pre-miR 100, anti-miR 100 or their respective controls is shown in Figures 3 and 4. We observed a decrease in protein levels, ranging from 30.8% to 49.6% in RT4 cells and 22.5% to 57.1% in T24 cells. The largest decreases in expression were observed for SMARCA5 (49.6%) and FGFR3 (40.5%) in RT4 cells and mTOR (57.1%), SMARCA5 (51.7%) and FGFR3 (46.9%) in T24 cells.

For RT4 cell line there was no difference in cell proliferation comparing transfected cells with pre-miR 100, anti-miR-100 and their negative controls. In T24 cell line exposed to pre-miR 100 44.5% of the cells were in G2/S or M versus 36% in the control. Although this difference was not statistically different (p = 0.37), there was an increase of 8.5% in cell proliferation after miR-100 transfection. There was no differences in the number of cells in apoptosis in both cell lines after miR-100 transfection. However, when T24 cells were exposed to anti-miR 100 there was an increase of 13% in cells in apoptosis, although this difference was not significant (p = 0.642) (Table 1).

**Table 1:** **Percentage of cells in phases G1, S and G2 / M and apoptosis in RT4 and T24 cell lines examed by flow cytometry**

	**miR-100**	**miR-control**	**Anti-miR-100**	**Anti-miR-control**
**Cell Line RT4**				
**G1(%)**	58	61	58	52
**S(%)**	8	7	8	8
**G2/M(%)**	32	32	34	34
**Apoptosis(%)**	57	59	60.5	62
**Cell Line T24**				
**G1(%)**	56.5	64	59	59
**S(%)**	12.5	6	8	9
**G2/M(%)**	32	30	33	32
**Apoptosis(%)**	62	64	39	26

The DNA ploidy is usually expressed as the DNA index (DI), that is, the ratio G0/G1 peak mean fluorescence of the tumor and the G0/G1 peak of the reference diploid (2n) population. There was no alteration in DNA ploidy in cell lines RT4 and T24 after transfection of pre-miR 100 and anti-miR 100.

## Discussion

This study investigated the regulation of putative target genes of miR-100 in two bladder urothelial cancer cell lines representatives of low (RT4) and high (T24) grade tumors. After miR-100 transfection, we observed a reduction in mRNA expression of the genes of interest, but the reduction was significant only for mTOR, BAZ2A and SMARCA5. Declines in protein levels were also detected.

The functional significance of miR-100 in cancer biology is supported by recent studies that show change in its expression in several types of cancer. Downregulation of miR-100 has been described in several tumors, such as squamous cell carcinoma of the tongue and oral cavity [6,7], nasopharyngeal cancer [8], hepatocellular carcinoma [9], hepatoblastomas [10] and serous ovarian carcinoma [11]. Overexpression of miR100 has been observed in medulloblastomas [12], and gastric [13], pancreatic [14] and prostate cancers [15].

In bladder urothelial cancer, our group and others have shown that the downregulation of miR-100 is characteristic of low-grade, non-invasive carcinomas and is thought to be an alternative pathway to the FGFR3 mutation typical of this type of tumor [16,17]. FGFR3 is a putative target of miR-100 and was previously shown to downregulate the gene expression by Luciferase reporter assay [18]. In our assays, we observed a reduction in FGFR3 mRNA expression of 20.6% in RT4 cells and 28% in T24 cells, which was not statistically significant compared to controls. However, there was a reduction in the protein expression by 40.5% in RT4 cells and 46.9% in T24 cells, leading us to conclude that miR-100 plays a role in regulating FGFR3 expression in both low and high grade urothelial carcinomas. FGFR3 mutation is the most common phenomenon in low-grade non-invasive urothelial carcinomas, described in 70% of the cases and identified as a molecular marker of non-aggressive disease [19,20]. Catto was the first to associate miR-100 loss and upregulation of FGFR3 in low-grade bladder cancer, proposing that miR-100 loss could precede the FGFR3 mutation [18]. In this context, miR-100 could be considered as a tumor suppressor miRNA because it regulates the activity of the tyrosine kinase receptor FGFR3.

The most affected genes in both types of cells after miR-100 transfection were SMARCA5 and mTOR. There was a significant reduction in the expression of both mRNA and protein.

mTOR (mammalian target of rapamycin) is a serine-threonine protein kinase, which acts with PI3K, AKT and the tumor suppressor gene PTEN to form a signaling pathway involved in the regulation of protein synthesis, cell growth, proliferation, survival, apoptosis, and angiogenesis [21]. Deregulation of the mTOR pathway has been related to oncogenesis in several malignancies, including bladder cancer [22]. Our data show that the mTOR gene was downregulated by miR-100 in bladder cancer cell lines. There was a significant reduction in mTOR mRNA expression and a strong reduction of mTOR protein expression in the T24 Cell Line (57.1%). This result suggests again that miR100 is a tumor suppressor miRNA because it downregulates the important PI3K/AKt/mTOR pathway. The same result was recently described by Xu et al., who identified miR-100 as a suppressor of cell growth in human bladder cancer in part at least through repression of mTOR [23].

SMARCA5 (hSNF2H) is a member of the SWI/SNF family involved in both homologous and non-homologous end joining recombination required for efficient DNA repair [24-26]. In this context, miR-100 should act as an oncomiR because the downregulation of SMARCA5 could result in deficiencies in DNA repair that could promote chromosomal instability, a hallmark of high-grade bladder cancer.

Other genes are also putative targets of miR-100 and could be important in the bladder carcinogenesis process. The Thanatos*-*associated protein (THAP) DNA-binding domain is a conserved C2CH zinc-finger motif shared by a large family of cellular factors with functions associated with cell-proliferation and cell cycle control. The function of THAP2 is not well known, but THAP1 has been described as an inhibitor of the cell cycle, interfering with pRb/E2F [27]. Others suggest that THAP proteins could play a major role in targeting genes to promote transcription regulation through interactions with protein complexes associated with chromatin remodeling [28]. Another role of THAP2 is associated with apoptosis, which, together with the transcription repressor protein Par-4 (prostate-apoptosis-response-4; a pro-apoptotic factor that suppresses the expression of BCL2, an important inhibitor of apoptosis), facilitates the process of programmed cell death [29]. Therefore, repression of THAP2 by miR-100 could affect apoptosis in urothelial cancer. We detected a 31.4% reduction in THAP2 mRNA in RT4 cells and a 24.2% reduction in T24 cells, the reduction in THAP2 protein expression was 29.8% in RT4 cells and 22.5% in T24 cells. The T24 cell line is a high-grade urothelial carcinoma, where the loss of function of tumor suppressor genes, such as p53 and Rb, and chromosomal instability are typical. In this context, miR-100 would act as an oncomiR because it downregulates THAP2, a putative tumor suppression protein that is important for Rb function, apoptosis induction and DNA chromatin remodeling [27,30,31].

In RT4 cells, BAZ2A was lowered after miR-100 transfection. BAZ2A, also known as TIP5 and WALp3, functions with the SWI/SNF family member SNF2h to form the NoRC (nucleolar remodeling complex). This complex induces nucleosome sliding, which is important for transcription regulation [32].

It is reasonable to think that the down expression of miR-100 that we previously observed in low-grade, non-invasive bladder urothelial cancer is related to the lack of control of mTOR and FGFR3 and to the maintenance of genomic stability due to not interfering with SMARCA5. In more aggressive urothelial cancers, miR-100 was related to increase in cell proliferation and its inhibition promoted increase in apoptosis. The decrease in expression of SMARCA5 and BAZ2A that we also identified in this lineage may primarily be influencing chromosomal stability that is a characteristic of this tumor, nevertheless no change occurred after inhibition or transfection of the miRNA. The observation of the behavior in gene and protein expression in human cancer cell lines throughout miRNAs exposure would bring a new knowledge that will permit their use as tumor markers and targets for treatment.
